# Personality trait predictors of adjustment during the COVID pandemic among college students

**DOI:** 10.1371/journal.pone.0248895

**Published:** 2021-03-17

**Authors:** David C. Rettew, Ellen W. McGinnis, William Copeland, Hilary Y. Nardone, Yang Bai, Jeff Rettew, Vinay Devadenam, James J. Hudziak

**Affiliations:** 1 Department of Psychiatry, Vermont Center for Children, Youth, and Families, University of Vermont Larner College of Medicine, Burlington, VT, United States of America; 2 Vermont Department of Mental Health, Child, Adolescent, and Family Unit, Waterbury, VT, United States of America; 3 Wellness Environment, University of Vermont, Burlington, VT, United States of America; 4 Department of Health and Kinesiology, University of Utah, Salt Lake City, UT, United States of America; 5 Department of Rehabilitation and Movement Science, University of Vermont, Burlington, VT, United States of America; Aalborg University, DENMARK

## Abstract

Personality traits have been found to be related to a variety of health outcomes. The aim of this study was to examine how personality traits were associated with adjustment to the COVID pandemic in college students. The sample included 484 first-year university students (76% female) attending a northeastern university who completed the Big Five Inventory (BFI) personality assessment at the beginning of a semester that was disrupted by the COVID pandemic. Using a phone-based app, students completed daily ratings of mood, perceived stress levels, and engagement in a number of health promotion activities (exercise, mindfulness, adequate sleep, etc.) throughout the semester both before and after the onset of the pandemic (e.g., a within-person longitudinal design). Results, as expected, showed that mood and wellness indices generally declined during the COVID period, although stress levels actually decreased. Further, irrespective of COVID, improved mood, less perceived stress and greater participation in health promotion activities were significantly associated with a number of personality traits including neuroticism (lower), extraversion (higher), agreeableness (higher), and conscientiousness (higher). Of primary interest, mixed-effects models were used to test how major personality traits interacted with any changes in daily ratings from the pre-COVID to COVID period. Significant interactions terms were found suggesting differential impacts of the COVID epidemic for students with low versus high levels of particular traits. Higher levels of extraversion, for example, were found to be related to decreases in mood as the pandemic progressed in contrast to those with lower extraversion, for whom there was a slight increase in mood over time. These data support the conclusion that personality traits are related to mental health and can play a role in a person’s ability to cope with major stressful events. Different traits may also be more adaptive to different types of stressors.

## Introduction

The COVID19 pandemic has had a major negative impact on mental health. Its effect was first documented in China but recently has been documented more globally [1, 2]. According to an April 2020 survey, more than a quarter of American adults meet criteria for “serious mental distress,” an eightfold increase since 2018 [3]. Data from the Healthline Mental Health Index indicates that up to 45% of US adults have elevated levels of depression and anxiety [4]. Not surprisingly, college students may be particularly vulnerable to the mental health effects of the COVID pandemic given the magnitude of the stressor during a period characterized by still developing emotional regulatory networks in the brain [5, 6]. A recent study that surveyed students across 195 U.S. colleges found that 71% of the sample reported increased anxiety due to the pandemic [7].

While major personality traits are not inherently good or bad, general patterns have been found between levels of traits and a number of outcomes such as overall wellness, happiness, educational and financial success, and mental health problems [8]. In general, more positive outcomes have been associated with high levels of extraversion, agreeableness, and conscientiousness and lower levels of neuroticism [9–11]. Similar patterns of associations have been found when it comes to resilience and coping after stressful events [12]. One recent meta-analysis examining the link between personality traits and mental health found that high levels of extraversion were most closely aligned with general “coping” [13]. In studies with children, temperament dimensions related to improved regulatory abilities (similar to the trait of conscientiousness) and reactivity (similar to the trait of neuroticism) have been found to buffer the impact of high-risk environments [14, 15]. Indeed, a personality profile that includes a lower level of anxiety and worry and higher levels of persistence and ambitiousness has been deemed the “resilient” profile in a study of medical students [16].

In studying the associations between personality traits and response to stressors, however, it is important not to assume that certain traits are universally more adaptive for all types of stressful circumstances. When it comes specifically to the COVID pandemic, there may be some relatively unusual qualities of this particular stressor that could lead to different traits being associated with better adjustment than what is typically found. High levels of extraversion, for example, which describe people who prefer increased levels of stimulation and social contact, could well find the mandated isolation and reduction in activities to be particularly difficult. Similarly, individuals with high levels of neuroticism might generally be less troubled with being confined at home and not having to navigate the everyday stressors of “normal” life, although this effect could be counterbalanced by increased worries about contracting the virus.

To examine how personality traits may be related to adjustment during the pandemic, the present investigation took advantage of a disruption in a study that was prospectively following a group of college students at a northeastern university. While the intent of the overall study was to examine the emotional health and wellness of students on campus, the COVID pandemic forced students to leave the university mid-semester. Consequently, a battery of mental health measures was collected in January 2020 prior to the epidemic (which will be referred to as pre-COVID). Students also completed daily mental health ratings using a smartphone app that began at the start of the semester in January and continued after mid-March until May when students were sent home (referred to as the COVID period).

We hypothesized that, similar to many previous studies, major personality dimensions would be associated with indicators of mental health pre-COVID in the typical pattern of better mental health being related to lower levels of neuroticism and higher levels of extraversion, agreeableness, and consciousness. Regarding the change in mental health indicators between pre-COVID and the COVID period, however, we predicted students high in extraversion would have a poorer adjustment to the pandemic relative to their less extraverted peers. We also predicted that many students with high neuroticism would not find the COVID period to be more stressful compared to the pre-COVID period. To our knowledge, this is the first study to examine the relations between personality traits and mental health functioning specifically through the COVID pandemic using a within-person longitudinal design.

## Materials and methods

### Participants

Eligible subjects for this study were first-year students in the 2019–2020 academic year at the University of Vermont who were willing to participate in yearlong study examining wellness activities, risk behaviors, and mental health. Students were recruited via on-campus posters, in-class presentations, and social media. Approximately two-thirds of these students were enrolled in the UVM Wellness Environment (WE), a residential and educational program designed to encourage students to make healthy decisions and limit substance use [17]. Inclusion criteria also included age between 18 and 25 and having an IPhone 5 or newer in order to complete assessments via an app. This study was approved by the University of Vermont’s IRB and all subjects provided written consent. Additional description of the sample can be found elsewhere [18].

**Fig 1:**
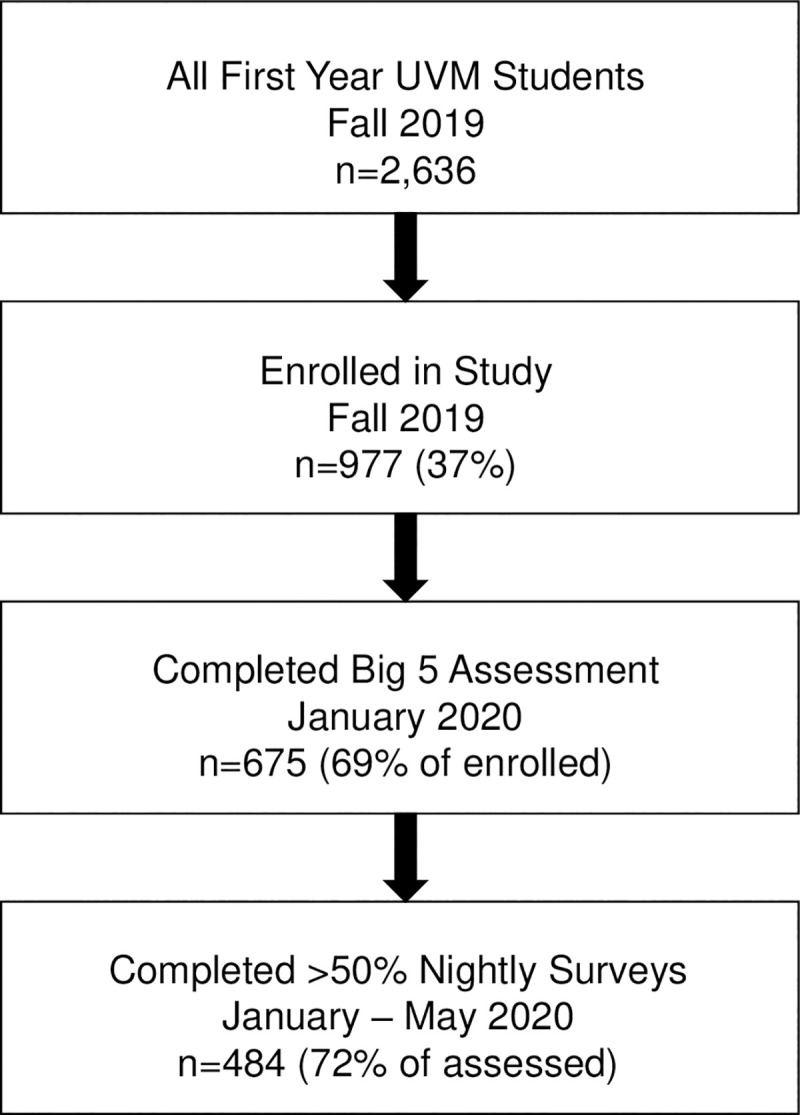
Study enrollment and attrition.

As mentioned, students completed a baseline assessment at the start of the spring semester in January 2020 and began doing their brief daily check-ins using their phones. By mid-March, the COVID pandemic had struck the northeastern United States and students were told on short notice to leave the university, return home, and finish their semester through remote online learning. Most did not have an opportunity to take all of their belongings with them. Despite these changes, participants continued to complete their daily mental health ratings by phone through the end of the semester in May (see Fig 1). Of the 675 students who completed the beginning of the spring assessment, 485 (84%) first-year students completed at least 50% of the nightly surveys across the spring semester. Those that routinely completed the daily surveys were not different in terms of gender, race/ethnicity, age, WE status, or subjective social status from those that did not.

### Measures

#### Personality traits

*Big Five Inventory (BFI)*. The BFI is a 44-item self-report personality scale based on Five-Factor Model of personality [19, 20]. Participants rate their agreement of each item along a 5-point Likert scale. The scale assesses five primary dimensions of personality, namely neuroticism, extraversion, openness, agreeableness, and consciousness. Neuroticism refers to the degree of emotional sensitivity and a tendency to experience negative emotions like fear and sadness. Extraversion describes those who like to be active, outgoing, and around other people. The dimension of openness relates to being creative and receptive to new experiences and ideas, while agreeableness captures a person’s level of compassion for others and willingness to cooperate in groups to avoid conflict. Conscientiousness, finally, refers to someone’s level of goal orientation, regulatory abilities, and organization.

The BFI has been shown to have good psychometric properties and strong reliabilities with longer personality instruments that utilize a similar “Big Five” framework [21, 22]. For this study, The BFI was administered to all participants at the beginning of the semester, pre-COVID.

#### Mental health and wellness

Participants each day gave a daily rating of their overall mood and stress level on a slider that was converted to a score of between 0 and 10. This information was collected from an app that was open daily from 7pm to 11:59pm. A daily wellness score, reflecting the level of participation in five healthy behaviors (i.e., minutes of exercise, limiting screen time, nutritional quality of meals, hours of sleep, and amount of water consumed) was also calculated by first dichotomizing the responses to indicate absence or presence of the wellness behavior and then summed to get an overall index. Each of the three daily survey measures (mood, stress, and wellness) was standardized for comparability. Of the 675 students who completed the beginning of the spring assessment, 485 (72%) first-year students completed at least 50% of the nightly surveys across the spring semester. Those that routinely completed the daily surveys were not different in terms of gender, race/ethnicity, age, WE status, or subjective social status from those that did not.

### Analysis

**Fig 2:**
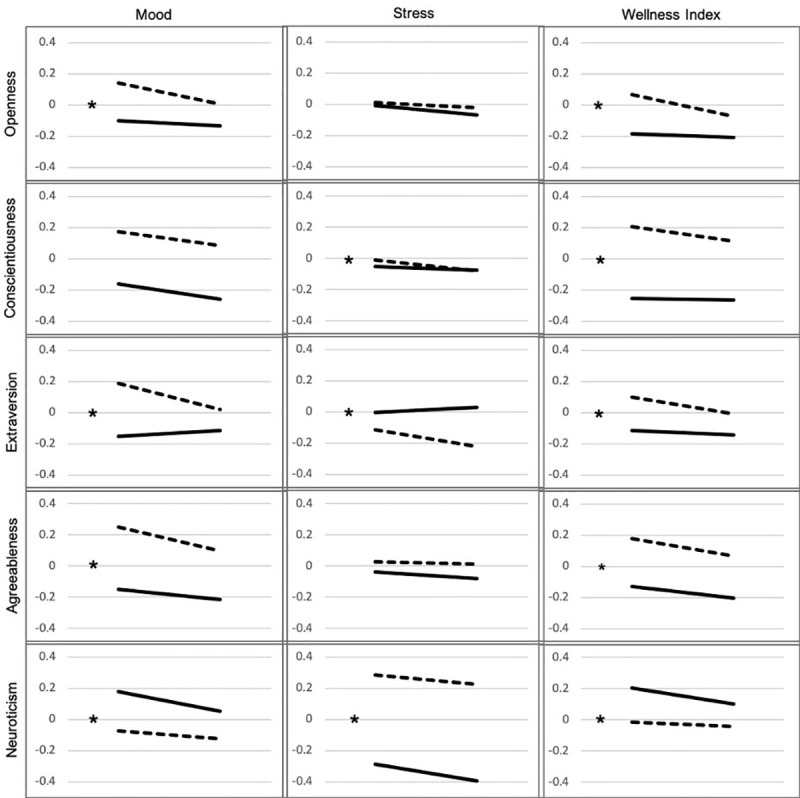
Mental health indicators from pre-COVID to COVID by students with high or low levels of Big 5 personality traits. Visualizations of Interactions using only extreme groups of standardized personality traits (y axis: low-solid lines; high-dashed lines) by COVID status (x-axis: before versus during)* = statistically significant interaction.

A series of mixed-effect, or multilevel, regression models were tested with MIXED in SPSS 26 to predict standardized scores for mood, stress, and wellness behavior. These models included a random intercept to account for repeated, correlated observations within individuals of daily survey items. Each outcome was predicted from baseline personality scores, COVID Status (before vs. during), and an interaction term between personality and COVID status. Covariates included enrollment in the wellness program, age, sex, subjectively rated socioeconomic status, semester week, and race/ethnicity. All analyses included the full range of personality scores. To illustrate findings visually in the figures only (see Fig 2), subjects with high or low levels of each trait (defined as being 0.6 standard deviations above or below the mean) were grouped together.

## Results

As mentioned, a total of 484 students (76% female) completed the personality measures at the beginning of the semester and had sufficient numbers of daily mental health ratings pre-COVD and during COVID after being sent home. The sample was over 90% white. Table 1 summarizes other information about study participants.

**Table 1:** Demographic information.

	N or Mean	% (Valid %) or SD
Gender		
Female	367	75.8
Male	117	24.2
Age	18.08	0.3
WE status		
WE	327	67.6
Non-WE	157	32.4
SES		
1–4	29	6
5–7	323	66.8
8–10	131	27.2
BFI		
Neuroticism	3.00	.75
high	140	28.9 (52.2)
low	128	26.4 (47.8)
Extraversion	3.11	.78
high	140	28.9 (50.4)
low	138	28.5 (49.6)
Openness	3.44	.61
high	117	24.2 (50.9)
low	113	23.3 (49.1)
Agreeableness	4.03	.60
High	181	37.4 (61.8)
low	112	23.1 (38.2)
Conscientiousness	3.82	.59
High	179	37.0 (62.6)
low	107	22.1 (37.4)

WE = participation in Wellness Environment program; BFI = Big Five Inventory; SES = Socioeconomic status.

### Personality main effects

Across the entire study period, all 5 major personality traits were found to be significantly related to mood and engagement in healthy activities (Table 2). Better mood and more wellness engagements was found to be significantly associated with lower levels of neuroticism and higher levels of extraversion, openness, agreeableness, and conscientiousness. Lower levels of perceived stress were also significantly related to higher neuroticism and higher extraversion scores.

**Table 2:** Personality main effects.

Traits	Mood			Stress			Wellness Engagement		
	b(SE)	p	95% CIs	b(SE)	p	95% CIs	b(SE)	p	95% CIs
Neuroticism	**-.13(.03)**	**< .001**	**-.12- -.06**	**.23(.03)**	**< .001**	**.17-.30**	**-.10(.03)**	**.002**	**-.17- -.03**
Extraversion	**.09(.03)**	**.008**	**.02-.16**	**-.07(.03)**	**.029**	**-.13- -.01**	**.07(.03)**	**.020**	**.01-.13**
Openness	**.10(.04)**	**.008**	**.03-.18**	.05(.04)	.200	-.02-.11	**.08(.04)**	**.023**	**.01-.15**
Agreeableness	**.16(.04)**	**< .001**	**.08-.22**	.03(.03)	.373	-.04-.10	**.14(.04)**	**< .001**	**-.09- -.03**
Conscientiousness	**.15(.04)**	**< .001**	**.07-.22**	.01(.03)	.825	-.06- .07	**.19(.03)**	**< .001**	**.12-.26**

Statistically significant results are shown in **bold** for personality traits (continuous z scores). E = Estimation; SE = Standard Error. 95% CIs = 95% Confidence Intervals. Covariates included COVID pandemic (before vs during), enrollment in the wellness program, age, sex, subjective social status, semester week, and race/ethnicity.

### Time main effects

As expected, daily ratings of mood [b = -.09 (SE = .01), p < .001, 95% CIs = -.12- -.07] and wellness engagement [b = -.07(SE = .01), p < .001, 95% CIs = -.10- -.04] also significantly declined after students were sent home from college because of the COVID pandemic. Perceived stress levels, however, significantly decreased [b = -.04 (SE = .02), p = .006, 95% CIs = -.08- -.01].

### Interactions

The primary focus of this study was to test the hypothesis that changes in mental health and wellness engagement from the pre-COVID to COVID period would be dependent upon levels of various personality traits. This hypothesis was confirmed with the trait X time interaction effect being statistically significant in 11 of the 15 tests performed (Table 3). Specifically, the decrease in mood that was generally experienced during the COVID period was less pronounced for those with higher levels of neuroticism and lower levels of extraversion, openness, and agreeableness, i.e. those who tended to have lower mood scores pre-COVID. The magnitude of these interaction effects, however, tended to be quite modest with the exception of extraversion. Here, students with higher levels of extraversion experienced a decline in mood over time while students with lower levels of extraversion showed a slight elevation. For wellness engagement, a similar pattern emerged in which those who tended to have less participation overall (those with higher levels of neuroticism and lower levels of extraversion, openness, and conscientious tended) maintained their relatively low levels of wellness engagement while those with low neuroticism and higher levels of extraversion, openness and conscientiousness saw steeper declines in engagement. Fig 2 shows extreme groups (.06 standard deviations below and above the mean) to better visualize continuous interaction results.

**Table 3:** Interactions between primary personality traits and the COVID pandemic.

Traits	Mood			Stress			Wellness Engagement		
	b(SE)	p	95% CIs	b(SE)	p	95% CIs	b(SE)	p	95% CIs
Neuroticism	**.03 (.01)**	**< .001**	**.02- .04**	**.02(.01)**	**.011**	**.00-.03**	**.02(.01)**	**< .001**	**.01-.04**
Extraversion	**-.07(.01)**	**< .001**	**-.08- -.06**	**-.04(.01)**	**< .001**	**-.06- -.03**	**-.02(.01)**	**< .001**	**-.04- -.01**
Openness	**-.04(.01)**	**< .001**	**-.05- -.03**	.02(.01)	.058	-.00-.03	**-.04(.01)**	**< .001**	**-.05- -.02**
Agreeableness	**-.03(.01)**	**< .001**	**-.04- -.02**	.01(.00)	.333	-.00-.02	**-.02(.01)**	**.020**	**-.03- -.00**
Conscientiousness	-.00(.01)	.482	-.02- .01	**-.02(.01)**	**.014**	**-.03- -.00**	**-.02(.01)**	**< .001**	**-.04- -.01**

Statistically significant results are shown in **bold** for interactions of personality traits (continuous z scores) by COVID status (before vs during). E = Estimation; SE = Standard Error. 95% CIs = 95% Confidence Intervals. Covariates enrollment in the wellness program, age, sex, subjective social status, semester week, and race/ethnicity in addition to main effects.

For perceived stress levels which, as previously mentioned, tended to *decrease* with time from the pre-COVID to COVID period, several significant interactions were also found. Students with high levels of extraversion experienced a decrease in stress levels in contrast to their less extraverted peers who reported a slight increase in stress. Smaller interactions were also found for neuroticism and conscientiousness.

## Discussion

This study aimed to examine the link between major personality dimensions and change in mental health functioning through the COVID pandemic in a group of college students. We utilized daily ratings of subjective mood, stress levels, and engagement in wellness activities such as mindfulness, healthy eating, and exercise obtained from a smartphone app that started before students were sent home due to COVID concerns and then continued as they completed the semester at home.

As has been reported in many studies examining the association between personality traits and various indices of well-being and functioning, we found robust associations with many of the higher-order personality traits. Lower levels of neuroticism and higher levels of extraversion were generally found to be related to improved mood, lower stress levels and more engagement in healthy activities. Higher levels of openness, agreeableness, and conscientiousness were also significantly related to better mood and more wellness engagement but were not significantly related to stress levels. Some of these significant main effects need to be interpreted with caution given the interactions found for some of these traits as levels changed between the pre-COVID and COVID period. High levels of agreeableness and conscientiousness showed particularly strong associations with better mood across the study period while higher levels of neuroticism were prominently and expectedly related to higher levels of perceived stress. High conscientiousness was significantly related to more participation in wellness activities and healthy activities. Conscientiousness refers to the tendency to be both goal oriented and to abilities that help people obtain goals such as reliability and organization. As such, this trait has often been associated with better health and well-being [23] and has been called the “most valuable psychological asset” a person can have when it comes to major personality traits [10].

The COVID pandemic also appeared to have a negative impact on our mental health indicators, although not as uniformly as we had expected. Comparing ratings between the pre-COVID and COVID period, mood and engagement in wellness activities generally decreased. Stress levels, however, significantly *fell* across the study period, although the magnitude of this drop was quite modest. Such a drop was hypothesized for students high in the trait of neuroticism but was found more globally. These results underscore how stressful college life can feel for some students [24]. It also may suggest some independence between mood and stress, the latter of which may be described more closely as anxiety. While mood and anxiety levels typically are found to move together–so much so that the two areas in combination are considered the core of “internalizing” problems [25], the COVID pandemic may present an important example of conditions that can separate the two domains. The college experience for many students provides engagement and relationships but, as has been well documented of late, can also be quite challenging and anxiety provoking [26]. With many exceptions, it is possible that the college environment, for those who do not find the experience overwhelming, represents an example of “good stress” that might contribute to a higher mood [27]. The COVID pandemic brought more isolation and inactivity but also some relief from the regular stresses of this environment and this may have overall depressed both mood and perceived levels of stress. Of course, it is possible that a more prolonged duration of disruption in regular life from COVID, as is now occurring, will result in perceived stress levels to rise. Further investigations with this sample and others will help reveal the long-term impact of COVID on experienced stress levels.

For the most part, associations between personality traits and mental health/wellness were preserved across the time period from before the COVID pandemic in January 2020 through the end of the semester in May. As previously mentioned, however, some interactions effects were found, suggesting a differential response to the COVID epidemic based on levels of specific traits. The dimension of extraversion was especially involved, with significant interaction effects found for mood, stress, and wellness participation. For mood, we found that those with higher levels of extraversion experienced a decrease in their mood as the COVID period progressed while mood ratings among those with lower levels of extraversion rose slightly. When it came to stress levels, however, those in the high extraversion groups felt decreased stress during the COVID period while those with lower extraversion experienced slightly more. There isn’t a straightforward interpretation of this combination of findings, but we speculate that, as hypothesized, more extraverted people might find the stimulation and challenges of busy academic life to be more rewarding. Leaving this environment for home isolation thus could have resulted in feeling less stressed but more bored and lonely, resulting in a decrease in mood. This finding partially supported our hypothesis, although it is important to note that students with higher levels of extraversion, despite having a clear decrease in mood with COVID, still reported an overall more positive mood than their low extraversion peers.

A similar, though less pronounced, pattern was found with neuroticism in which those with lower levels experienced a larger drop in mood levels with COVID compared to students with high neuroticism (although the higher neuroticism group continued to report lower mood overall). It is possible that this finding comes from a “floor effect” as students with higher levels of neuroticism had a lower mood at baseline. Alternatively, it is possible that the disruption from COVID represented a relatively bigger “loss” for those with lower levels of neuroticism.

These findings need to be interpreted with appreciation for some of the limitations of this study. First, these data were obtained from college students at a single university. As such, the generalizability of this study to other populations may be limited. Secondly, it is also not possible to prove with these data that is was the COVID pandemic and not another unmeasured factor that was responsible for any changes in the mental health ratings. Small but meaningful changes in personality have been documented in college students under “normal” conditions, although this has generally been found to occur over longer periods of time [28]. Finally, we note that both the personality and mental health assessments were obtained from self-report.

This study, however, also has some noticeable strengths. The use of daily ratings of our mental health indices may have provided a more valid assessment of these parameters over time and minimized bias inherent in more retrospective reporting. Personality assessment was also performed using a well-validated instrument based on a widely accepted and researched personality model.

## Conclusion

In summary, this study focused on changes in mood, perceived stress levels, and engagement in healthy behaviors in a group of college students as the COVID pandemic forced this group to leave campus. Personality traits assessed prior to this stressor were found to be strong predictors of these mental health indices both prior to and during the pandemic. While many of these associations are consistent with prior research on personality and mental health, we also found indications that the COVID pandemic presents some challenges to groups with specific personality characteristics, such as high levels of extraversion, that typically are associated with increased resilience. While more research to understand the relations between personality dimensions and adjustment to the COVID pandemic and other stressors is needed, these data suggest a more complicated picture when it comes to how various personality traits may provide risk or buffers when it comes to coping with major stressors.
