# Correction: Personality trait predictors of adjustment during the COVID pandemic among college students

**DOI:** 10.1371/journal.pone.0259431

**Published:** 2021-10-27

**Authors:** David C. Rettew, Ellen W. McGinnis, William Copeland, Hilary Y. Nardone, Yang Bai, Jeff Rettew, Vinay Devadanam, James J. Hudziak

The seventh author’s name is spelled incorrectly. The correct name is: Vinay Devadanam.
